# Alterations of the Human Lung and Gut Microbiomes in Non-Small Cell Lung Carcinomas and Distant Metastasis

**DOI:** 10.1128/Spectrum.00802-21

**Published:** 2021-11-17

**Authors:** Hui Lu, Na L. Gao, Fan Tong, Jiaojiao Wang, Huanhuan Li, Ruiguang Zhang, Hong Ma, Nong Yang, Yongchang Zhang, Ye Wang, Zhiwen Liang, Hao Zeng, Wei-Hua Chen, Xiaorong Dong

**Affiliations:** a Cancer Center, Union Hospital, Tongji Medical College, Huazhong University of Science and Technology, Wuhan, China; b Key Laboratory of Molecular Biophysics of the Ministry of Education, Hubei Key Laboratory of Bioinformatics and Molecular-Imaging, Department of Bioinformatics and Systems Biology, College of Life Science and Technology, Huazhong University of Science and Technology, Wuhan, China; c Department of Medical Oncology, Hunan Cancer Hospital/The Affiliated Cancer Hospital of Xiangya School of Medicine, Central South University, Changsha, China; d Institution of Medical Artificial Intelligence, Binzhou Medical University, Yantai, China; Wayne State University

**Keywords:** gut microbiota, lung microbiota, machine learning, patient stratification, NSCLC, distant metastasis, brain metastasis

## Abstract

Non-small cell lung cancer (NSCLC) is the leading cause of cancer-related deaths worldwide. Although dysbiosis of the lung and gut microbiota have been associated with NSCLC, their relative contributions are unclear; in addition, their roles in distant metastasis (DM) are still illusive. We recruited in total 121 participants, including 87 newly diagnosed treatment-naive NSCLC patients of various stages and 34 healthy volunteers, and surveyed their fecal and sputum microbiota. We compared the microbial profiles between groups, identified microbial biomarkers, and generated machine learning models for distinguishing healthy individuals from patients with NSCLC and patients of various stages. We found significant perturbations of gut and sputum microbiota in patients with NSCLC and DM. A machine learning model combining both microbiota (combined model) performed better than an individual data set in patient stratification, with the highest area under the curve (AUC) value of 0.896. Sputum and gut microbiota both contributed to the combined model; in most cases, sputum-only models performed similar to the combined models. Several microbial biomarkers were shared by both microbiotas, indicating their similar roles at distinct body sites. Microbial biomarkers of distinct disease stages were mostly shared, suggesting biomarkers for DM could be acquired early. Furthermore, Pseudomonas aeruginosa, a species previously associated with wound infections, was significantly more abundant in brain metastasis, indicating that distinct types of DMs could have different microbes. Our results indicate that alterations of the sputum microbiota have stronger relationships with NSCLC and DM than the gut and strongly support the feasibility of metagenome-based noninvasive disease diagnosis and risk evaluation. (This study has been registered at ClinicalTrials.gov under registration no. NCT03454685).

**IMPORTANCE** Our survey on gut and sputum microbiota revealed that both were significantly disturbed in non-small cell lung cancer (NSCLC) and associated with distant metastasis (DM) while only the sputum microbiota was associated with non-DM NSCLC. The lung microbiota could therefore have a stronger association with (and thus may contribute more to) disease development than the gut microbiota. Mathematic models using both microbiotas performed better in patient stratification than using individual microbiota. Sputum models, however, performed similar to the combined models, suggesting a convenient, noninvasive diagnostic for NSCLC. Microbial biomarkers of distinct disease stages were mostly shared, suggesting that the same set of microbes were underlying disease progression, and the signals for distant metastasis could be acquired at early stages of the disease. Our results strongly support the feasibility of noninvasive diagnosis of NSCLC, including distant metastasis, are of clinical importance, and should warrant further research on the underlying molecular mechanisms.

## INTRODUCTION

Lung cancer (LC) is the leading cause of cancer-related deaths worldwide, with non-small cell lung cancer (NSCLC) being the most common form (1). Despite the recent development of therapies for NSCLC, tumor metastasis is the main cause of recurrence and mortality in patients with NSCLC (1). One of the key challenges is the low heritability of LC susceptibility revealed by genetic studies. Although numerous studies have established the important roles of somatic mutations as well as inheritable familial risks (2, 3), the genetic influence can only explain 3–15% of the heritability (4, 5), depending on the surveyed population.

Conversely, nongenetic factors, including lifestyles, environmental factors, and lung and gut microbes, are believed to be the main etiological factors of the disease. In particular, numerous recent studies have shown that both lung and gut microbiota are involved in the development of LC (6–8). For example, researchers have used samples from bronchoalveolar fluid, tissues, and spontaneous sputum of LC patients for bacterial identification and microbiome characterization (7, 9–11). The former two were invasively collected via bronchoscopy or surgery; however, the latter, which were easily accessible, provided a noninvasive method of obtaining lower respiratory tract samples. In addition, because sputum is secreted from bronchi and bronchioles of the lower respiratory tract, molecular changes detected in sputum could reflect those in the lower respiratory tract for greater detection sensitivity for LC. Huang et al. compared the microbiotas of the lung (bronchoalveolar fluid) and sputum in LC patients and found similarly dominant taxonomic entities (taxa) at both phylum (such as *Firmicutes*, *Bacteroidetes*, *Proteobacteria*, *Actinobacteria*, and *Fusobacteria*) and genus (such as Streptococcus, *Prevotella*, *Neisseria*, *Porphyromonas*, *Veillonella*, and Haemophilus) levels, although some taxa might show significantly different abundances between the two microbiotas (12). In addition, Zheng et al. compared the pathogens isolated from lung (bronchoalveolar fluid) and sputum and found that the top 10 most abundant ones were identical in both types of samples (13). Together, these studies suggest that sputum could be used as a close proxy for the lung.

Dysbiosis of the gut microbiome has also been associated with many cancers (8, 14–16), including LC (8). A previous study suggested an increase in *Enterococcus* in the stool of LC patients compared to that of healthy subjects and a decrease in *Bifidobacterium* and *Actinobacteria* (6), of which others have shown that the response to immunotherapy in NSCLC patients is associated with specific changes of individual species, such as Alistipes putredinis, Bifidobacterium longum, and Prevotella copri as well as the overall diversity of the gut microbiome (7, 8). Furthermore, increasing evidence has shown that the gut microbiome may play important roles in cancer by modulating inflammation (17) and host immune response (18, 19) and directly interacting with therapeutic drugs (20).

Despite these significant advances, two important questions remain. First, it is still unclear which microbiota has a stronger association with the development of NSCLC; the relative importance of local (i.e., lung-associated) versus gut microbiota has been recently discussed (21), but no direct evidence has been provided so far. Second, their alterations along with distant metastasis (DM) of NSCLC are yet to be characterized. To address these issues, we first conducted a comprehensive survey on both feces and sputum (as a proxy for lung) microbiota in NSCLC patients of various stages, including stage IV patients suffering from distant metastasis, and compared them with healthy controls of matching demographic and clinical characteristics. We then built mathematical models using the taxonomic profiles of both gut and sputum microbiota to test their ability to distinguish patients of different disease stages and from healthy controls and evaluate their relative contributions to the models.

## RESULTS

### Differential microbial diversity between sputum and gut microbiotas.

We enrolled in total 121 participants who completed our study protocol (see Materials and Methods), among which 87 were newly diagnosed with NSCLC who had not previously received any anticancer therapy nor were treated with any antibiotics (i.e., treatment naive), and 34 were healthy volunteers. We classified patients into distinct disease stages, that is, from I to IV according to the 8th American Joint Committee on Cancer guidelines (22). All subjects lived in Hubei Province, China. As shown in Table 1, we found comparable demographic and clinical characteristics of these subjects between the groups we were interested in. In this study, we used “Control,” “NSCLC,” “I_III,” and “DM” to refer healthy controls (Control), patients of all stages (NSCLC), patients of stages I to III (I_III), and patients with distal metastasis (DM, also referred as to stage IV), respectively.

**TABLE 1:** Clinical characteristics of healthy subjects and NSCLC patients

		Sputum						Feces			
					*P* value^*a*^					*P* value^*a*^	
		Healthy *n* = 30	Stage I to III *n* = 27	Stage IV *n* = 39	Healthy versus stage I to III	Stage I to III versus stage IV	Healthy *n* = 29	Stage I to III *n* = 30	Stage IV *n* = 55	Healthy versus stage I to III	Stage I to III versus stage IV
Age (yrs)											
Mean ± SD		54.67 ± 12.46	59.44 ± 6.807	58.31 ± 7.79	0.075	0.542	55.83 ± 12.04	59.17 ± 6.69	58.36 ± 8.292	0.197	0.650
Gender											
Male, *n* (%)		17 (56.67)	18 (66.67)	29 (74.36)	0.587	0.584	15 (51.72)	19 (63.33)	37 (67.27)	0.435	0.812
Female, *n* (%)		13 (43.33)	9 (33.33)	10 (25.64)			14 (48.28)	11 (36.67)	18 (32.73)		
BMI (kg/m^2^)											
Mean ± SD		22.34 ± 2.52	23.74 ± 3.80	22.13 ± 3.446	0.107	0.063	22.28 ± 2.38	23.58 ± 3.92	22.16 ± 3.161	0.141	0.061
Smoking status											
Smoker, *n* (%)		14 (46.67)	14 (51.85)	23 (58.97)	0.793	0.620	12 (41.38)	15 (50.00)	29 (52.73)	0.604	0.244
Nonsmoker, *n* (%)		16 (53.33)	13 (48.15)	16 (41.03)			17 (58.62)	15 (50.00)	26 (47.27)		
Disease stage											
Stage I, *n* (%)		—^*b*^	9 (33.33)	0	—	—	—	11 (36.7)	0	—	—
Stage II, *n* (%)		—	7 (25.93)	0	—	—	—	7 (23.3)	0	—	—
Stage III, *n* (%)		—	11 (40.74)	0	—	—	—	12 (40.0)	0	—	—
Stage IV, *n* (%)		—	0	39 (100.00)	—	—	—	0	55 (100.00)	—	—
Lung cancer pathology											
Adenocarcinoma, *n* (%)		—	19 (70.37)	26 (66.7)	—	—	—	22 (73.3)	40 (72.73)	—	—
Squamous cell carcinoma, *n* (%)		—	6 (22.22)	9 (23.1)	—	—	—	6 (20.0)	12 (21.82)	—	—
Non-small cell carcinoma, *n* (%)		—	2 (7.41)	4 (10.3)	—	—	—	2 (6.7)	3 (5.45)	—	—

a Statistical differences were calculated by independent-samples *t* tests for continuous data and χ^2^ tests for count data.

b —, indicates no data.

**FIG 1:**
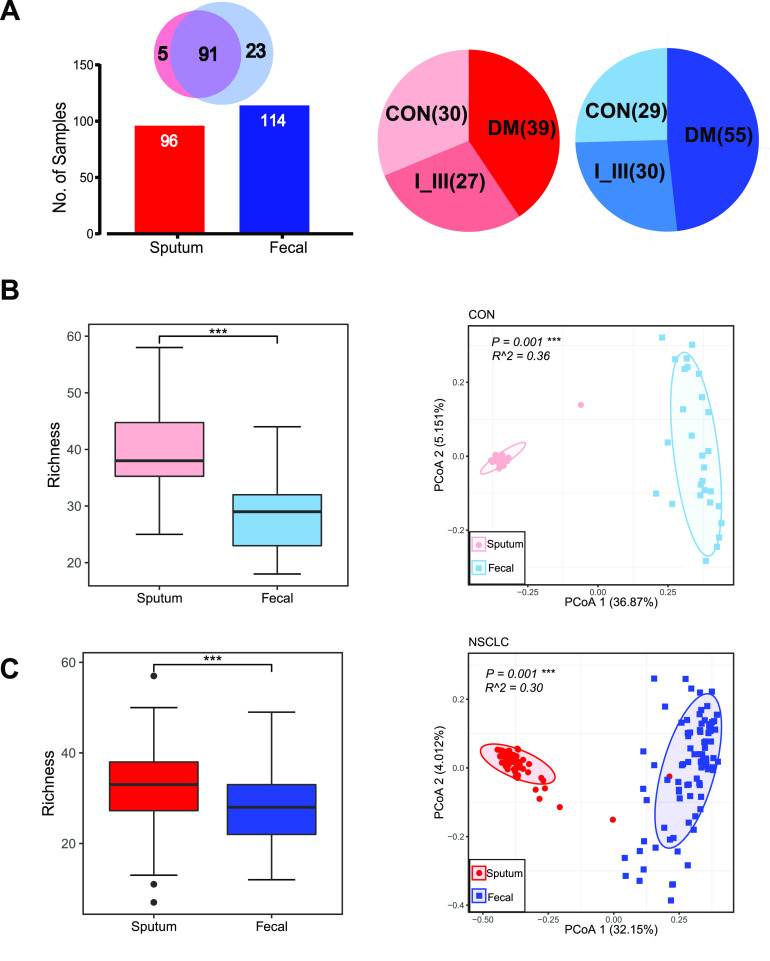
Sputum and gut microbiota differed significantly in terms of alpha- and beta-diversities. (A) Numbers of sputum (red) and gut (blue) samples collected in this study and their distributions in healthy controls and distinct disease stage groups; CON, healthy controls; I_III, patients with stages of I to III; DM, patients with distant metastasis (also referred to as stage IV). Disease stages were assigned according to the 8th American Joint Committee on Cancer guidelines. (B) Comparisons of alpha-diversity and beta-diversity between the sputum and gut in healthy controls. Richness index (alpha-diversity; left) at the genus level was significantly lower in feces; principal-coordinate analysis (PCoA; right) based on Bray-Curtis distance at the genus level showed that the overall microbiota composition was different between fecal and sputum samples. Wilcoxon rank sum tests were used to compare between groups. (C) Comparisons of alpha-diversity (left) and beta-diversity (right) between sputum and gut in NSCLC patients (stages I to IV). Level of significance: ***, *P < *0.001; **, *P < *0.01; *, *P < *0.05; NS, *P ≥ *0.05.

We collected in total 30 sputum and 29 fecal samples from the healthy controls and 66 sputum and 85 fecal samples from the patients (NSCLC) (Fig. 1A) and submitted them for 16S rRNA gene sequencing (see Materials and Methods). Using amplicon sequence variants (ASVs) to track the dynamics of the abundance of different bacterial phyla in sputum and feces, we found that there were twice as many bacterial phyla in sputum than in feces (Fig. S1A to C in the supplemental material). *Bacteroidetes* and *Firmicutes* are the most abundant phyla both in sputum and feces (Fig. S1A and B). The dominant phyla of sputum, such as *Bacteroidetes*, *Firmicutes*, *Proteobacteria*, *Actinobacteria*, and *Fusobacteria*, were also found in lung microbiota (12). Ten of eleven bacterial phyla in feces were shared with sputum, except for the phylum *Verrucomicrobia*, to which the well-known *Akkermansia* belongs (Fig. S1C). Although 4 of the 11 phyla unique to the sputum samples were related to oral cavities, they were of extremely low relative abundances. Therefore, sputum samples could be contaminated by oral microbiota, but their effects were negligible. We also checked the overlap of top-ranking taxa with high relative abundance between neighboring disease stages (Fig. S1D). For example, we found that 9 of the top 10 genera were shared in sputum between neighboring disease stages (Fig. S1D, top); *Capnocytophaga* decreased, and Pseudomonas increased in the sputum of DM patients. Similarly, seven out of the dominant genera (*Prevotella*, Streptococcus, *Veillonella*, *Neisseria*, Haemophilus, *Fusobacterium*, and *Porphyromonas*) in sputum were also in the lung microbiota (12). Unlike the sputum with more varied genera, there was only one main family in the gut, *Lachnospiraceae*, most members of which were found in the human or animal digestive tract (23). However, only one genus, Streptococcus, was shared by the sputum and gut. These results illustrated the vast difference between the sputum and gut microbiotas.

As shown in Fig. 1B and C and Fig. S2A and B, we found that the microbial diversity, as measured by Richness index, was significantly higher in sputum than in the gut in both healthy controls (Fig. 1B, left) and NSCLC groups (Fig. 1C, left) (Wilcoxon rank sum test). Furthermore, we found significant lower alpha-diversities (Fig. S2A, evenness index, top; Shannon index, middle; Simpson index, bottom) in the gut of healthy controls. We also performed a principal-coordinate analysis and an Adonis test based on Bray-Curtis distance at the genus level to assess the beta-diversity in microbial composition and found that the sputum microbiota was significantly different from the gut in healthy controls (Fig. 1B, right) and patients (Fig. 1C, right). Together, our results suggested that the sputum microbiota was significantly different from the gut microbiota and had significantly higher microbial diversity.

### Global alterations of sputum and fecal microbiotas in NSCLC patients of different stages.

**FIG 2:**
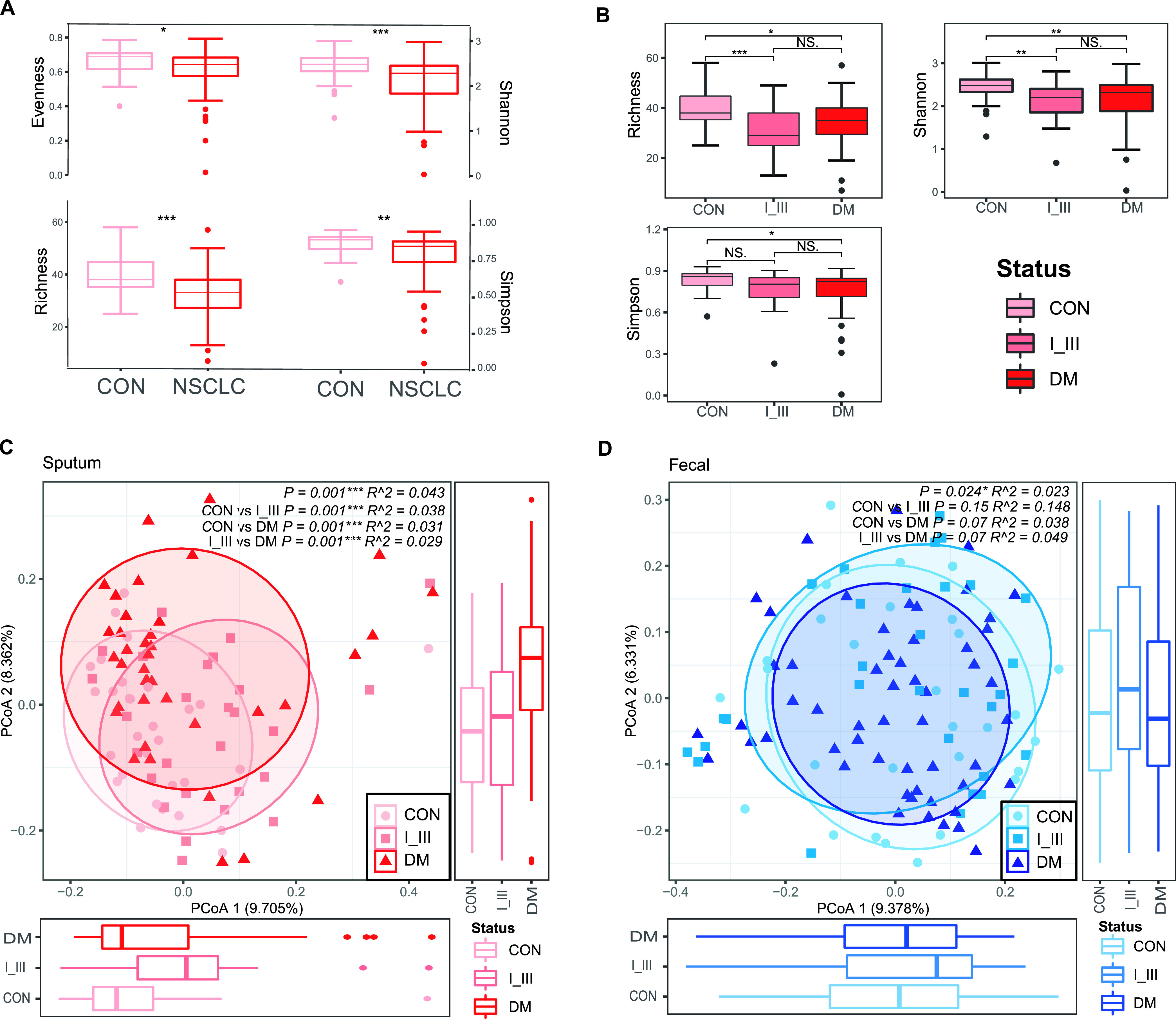
Global alteration of the sputum microbiota was associated with NSCLC and distant metastasis (A, B), while the fecal microbiota was only significantly associated with the latter (C, D). (A) Significant differences were found in alpha-diversity between healthy controls and individuals with NSCLC. Richness index, Shannon index, evenness index and Simpson index at the genus level were significantly lower in patients than in healthy controls. A Wilcoxon rank sum test was used to compare between groups. Level of significance: ***, *P < *0.001; **, *P < *0.01; *, *P < *0.05; NS, *P ≥ *0.05. (B) Alpha-diversity of sputum dysbiosis in pairwise comparisons. The richness index (top left), Shannon index (top right), and evenness index (bottom left) are shown. The Shannon index and richness index were significantly lower in patients than in healthy controls. An ANOVA with a *post hoc* Tukey HSD test was used to compare between groups. Level of significance: ***, *P < *0.001; **, *P < *0.01; *, *P < *0.05; NS, *P ≥ *0.05. (C) Significant differences were found in beta-diversity between controls and individuals with NSCLC as well as between controls and I_III, controls and DM, and I_III and DM groups, indicating that dysbiosis of the sputum microbiota was associated with lung cancer development and metastasis. Conversely, applying similar analyses to fecal samples, no differences in alpha-diversities were apparent (D), but the beta-diversity in controls versus DM and I_III versus DM were different (*P = *0.07), suggesting that fecal microbiota dysbiosis was associated with distal metastasis but not NSCLC.

We next investigated the global alterations (i.e., dysbiosis) of sputum and gut microbiotas in patients of different stages and between patients and healthy controls. As shown in Fig. 2A, we found significantly lower alpha-diversities (evenness index, top left; Shannon index, top right; richness index, lower left; Simpson index, lower right) in the sputum microbiota of NSCLC groups compared to Controls. We could find the same trend in I_III and DM groups (Fig. 2B; Fig. S3A). We also found significantly different beta-diversities between NSCLC and Control groups (*P = *0.001) (Fig. S3B) and pairwise comparisons (Fig. 2C). Pairwise differences between groups were assessed using the pairwise.adonis function adjusted for false-discovery rate (FDR) correction. Thus, the dysbiosis of the sputum microbiota was associated with the development and progression of NSCLC. More specifically, according to the *R*^2^ values, the maximum percent variances explained was only 4.3% even though the bacterial profiles of the sputum samples among patients of different stages were significantly different. This might be related to the various influences on the microbiota.

Conversely, in the gut microbiota, we did not find significant differences between NSCLC and Control groups (Fig. S3C to F) in neither alpha-diversities nor beta-diversities (Fig. 2D; Fig. S3G). It should also be noted that the maximum percent variances explained in fecal bacterial profiles was only 4.9%.

### A significant proportion of microbial biomarkers was shared by sputum and gut microbiotas.

**FIG 3:**
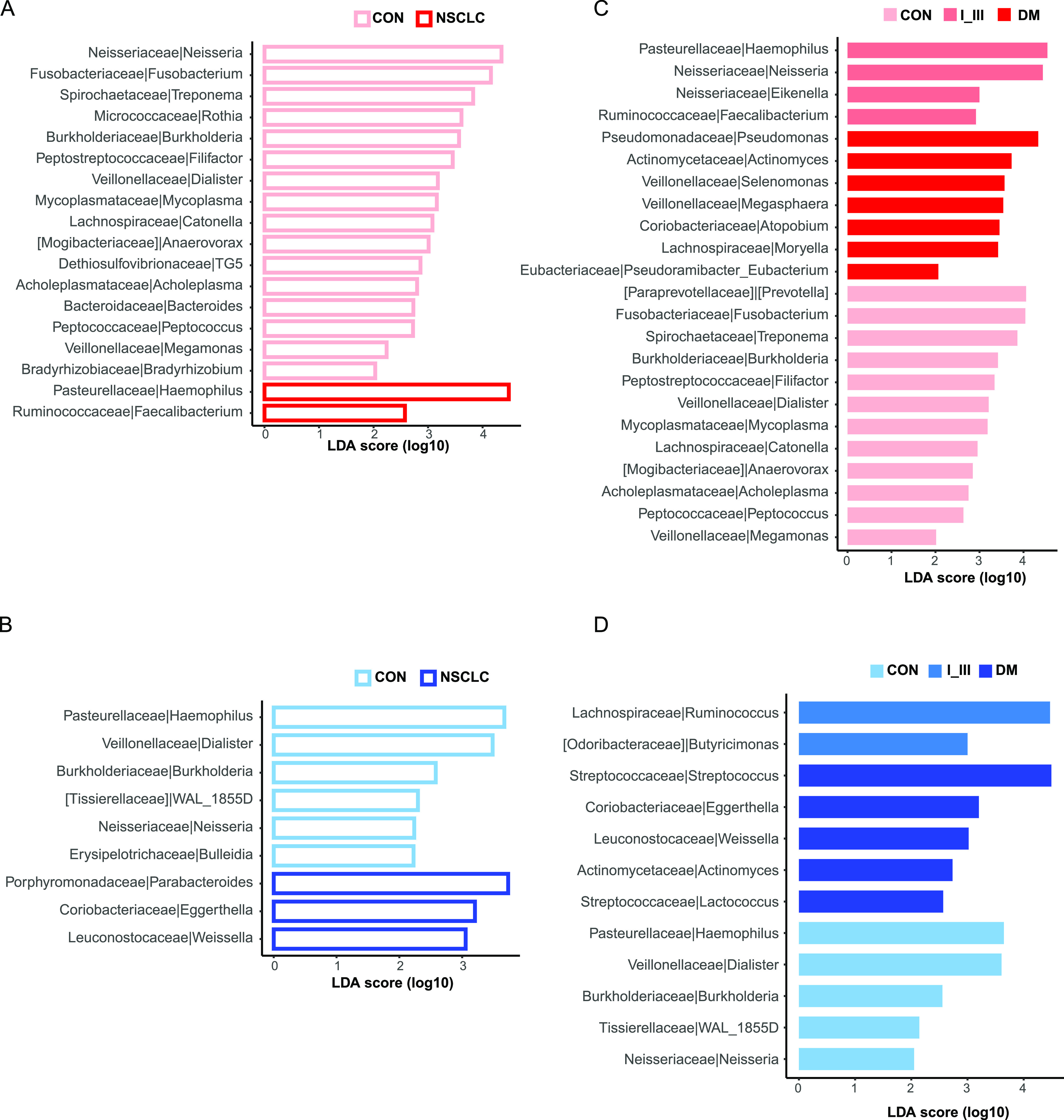
Shared and distinct microbial biomarkers between subject groups in sputum (red) and fecal (blue) microbiota. Differentially abundant microbial biomarkers between subject groups were identified using LEfSe analyses; red and blue bar plots indicate LEfSe results for sputum and fecal microbiota, respectively. The relative abundance of 18 and 9 genera was significantly different between NSCLC and control groups in sputum (A) and feces (B), respectively. To identify biomarkers for specific disease stages, we compared neighboring groups along the disease progression in sputum (C) and feces (D). LDA, linear discriminant analysis.

We searched for individual taxa that showed differential abundances between subject groups (i.e., microbial biomarkers) using linear discriminant analysis effect size (LEfSe) analysis (see Materials and Methods) and summarized the results in Fig. 3.

We first compared all NSCLC patients as a whole (i.e., from stages I to IV) against healthy controls. We found that 16 genera were depleted in sputum samples of the NSCLC group (Fig. 3A); both *Neisseria* and *Fusobacterium* were the dominant genera in the lung microbiota (13). These results confirmed that the sputum microbiome had been significantly altered in patients. Conversely, Haemophilus was the most enriched genus in sputum samples of the NSCLC group, most ASVs of which (∼77.3%) could be reliably identified as Haemophilus parainfluenzae and Haemophilus influenzae. Both of them are Gram-negative, facultatively anaerobic coccobacillus and have been previously identified in lung cancer patients with various degrees of lung inflammation (24). The whole of *Faecalibacterium* ASVs were identified as Faecalibacterium prausnitzii, which was highly abundant in the human gut microbiota in healthy individuals and can regulate the expression of proinflammatory and anti-inflammatory cytokines in the gut-lung axis through the secretion of extracellular alveoli (25).

We found that the genus *Eggerthella* from the family *Coriobacteriaceae* was significantly enriched in NSCLC samples of the gut (Fig. 3B). *Coriobacteriaceae* is a group of Gram-positive bacteria that are often nonmotile, nonspore forming, nonhemolytic, and strictly anaerobic (26). They are normal dwellers of mammalian body habitats, including the oral cavity, gastrointestinal tract, and genital tract (26). Consistent with our results, several members of the genera, including *Atopobium*, *Eggerthella*, *Gordonibacter*, *Olsenella*, and *Paraeggerthella*, had been implicated in the development of various clinical pathologies, including abscesses (27), periodontitis (28), intestinal diseases, and tumors (29, 30).

We next compared multiple groups along the disease progression (i.e., Control, I_III, to DM) to identify biomarker species for specific disease stages (Fig. 3C and D). We found that the family *Coriobacteriaceae* and the genus *Actinomyces* were enriched in the sputum and gut of DM patients. *Actinomyces* belongs to the normal resident microbiota, is considered to be a facultative pathogen, and is usually associated with the breakdown of normal physical barriers, such as the disruption of mucosal membranes (31). Most infections with *Actinomyces* are polymicrobial, and members of the Streptococcus genus are the most commonly associated organisms (32), which were also significantly increased in the gut of DM patients in our study. Streptococcus contains a variety of species, some of which cause disease in humans and animals. The ASV number of Streptococcus sanguinis and Streptococcus mutans accounted for 45% of the total ASVs of confirmed species using SPINGO. These Gram-positive cocci are a major inhabitant of the oral microbiota and are considered to be related to dental caries and tooth decay. Streptococcus acted synergistically by inhibiting host defense mechanisms and reducing oxygen tension in the affected tissue, which enhanced the growth of *Actinomyces* (32). In addition, we found that Pseudomonas associated with wound infections was highly abundant in the sputum of the DM group.

Together, our results suggested that a significant proportion of sputum and gut microbial biomarkers were shared; the overlap could be due to extensive transmission from the oral site to other body sites, as suggested previously (33), or to exposure to the same environment.

### The contributions of sputum and fecal microbiotas in patient stratification.

We next assessed the potential value of the sputum and gut microbiotas in patient stratification. We generated predictive models using the random forest algorithm implemented in SIAMCAT (34), evaluated the model performance with 10-times cross-validation, and reported the averaged area under receiving operating characteristic curves values (AUC; see Materials and Methods). We used 90% of the sputum and gut microbiota separately as the training set to generate the predictive models and then predicted and reported the AUC on the testing set (referred to as sputum and gut models, respectively). As shown in Table 2, we found that the sputum microbiota performed better than the gut in patient stratification (Table 2).

**TABLE 2:** The AUC values of classifying models

	Features	CON versus NSCLC^*a*^	CON versus I_III^*a*^	I_III versus DM^*a*^	CON versus DM^*a*^
Sputum	All	0.750	0.850	**0.767**	0.744
	DAM^*b*^	0.728	0.867	0.742	0.756
	RFE^*c*^	0.803	0.850	0.708	0.739
Gut	All	0.760	0.817	0.760	0.650
	DAM^*b*^	0.515	—^*d*^	—	—
	RFE^*c*^	0.692	0.717	0.673	0.720
Sputum + gut	All	**0.825**	**0.896**	0.754	**0.866**
	RFE^*c*^	0.783	0.854	0.691	0.804

a In each comparison (e.g., CON versus NSCLC), the modeling strategies with the highest and lowest performance as measured by AUC values are highlighted in bold font and gray shading, respectively.

b DAM, differentially abundant microbes. In more than 90% of tests, there were too few or no differentially abundant genera, so it was considered that the DAM model was not suitable for fecal microbiota.

c RFE, features with top-ranked contributions to the models were used (see Materials and Methods).

d —, indicates no data.

We then selected the most important genera and generated the predictive models via two methods: differentially abundant microbes (DAM) and recursive feature elimination (RFE). In DAM models, we first got the differentially abundant microbes from the training set using the check.associations function from the SIAMCAT package and then generated models and predicted on the testing set. RFE was an efficient approach because we were able to eliminate features from a training data set for feature selection, rank features by importance, discard the least important features, and refit the model for AUC reporting. As shown in Table 2, we found that both the sputum RFE and sputum DAM models could perform either slightly better than or comparable to the sputum model in the effect of prediction and performed much better than gut models in all subject group comparisons.

We next built predictive models using both the sputum and fecal microbiome data as input (referred to as “mixed models” below). Among the enrolled subjects, we identified 91 subjects who had both sputum and fecal samples, among which 26, 27, and 38 were healthy controls, stage I_III, and DM patients, respectively. As shown in Table 2, we found that the mixed model performed best in all subject group comparisons except the I_III versus DM group, in which the sputum model performed best (Table 2). These results suggested that the sputum microbiota contributed more than the gut microbiota in patient stratification. In most cases, the sputum-only microbiota alone was sufficient for decent model performance.

**FIG 4:**
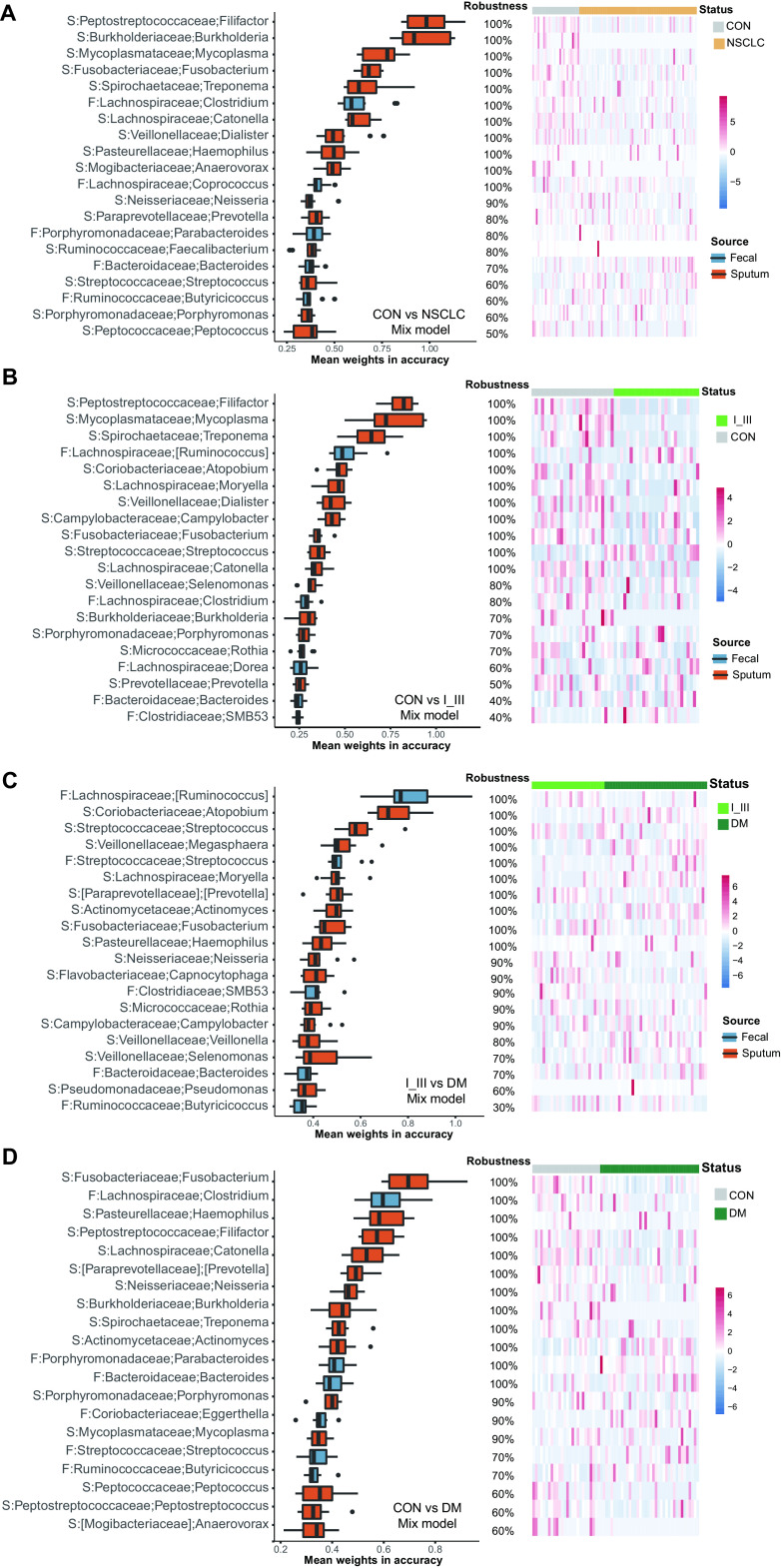
The top-ranked genera of the mixed models shown in Table 2 for each disease stage. The genera were ranked by the robustness of 1,000 repeats; therefore, box plots were used here to demonstrate the means and distributions of these values. (A) Control versus NSCLC. (B) Control versus I_III. (C) I_III versus DM. (D) Control versus DM. Red boxes represent sputum-derived genera, and blue boxes represent gut-derived genera. Please consult Table 2 for details on the model performance. F, feces; S, sputum.

We also checked the most important genera ranked according to their importance in mixed models to further confirm the contributions of the sputum and gut microbiotas, respectively (Fig. 4A to D; Fig. S4A to D). In the top 20 genera, sputum microbiota accounted for three-quarters, suggesting again that the sputum microbiota contributed more to patient classification. Most of the genera were of different abundance and shared in mixed models except a few genera, such as the genus Campylobacter, which was enriched in healthy controls and DM patients in sputum. People could get Campylobacter infections by eating undercooked poultry or raw foods, some of which usually recover on their own without antibiotic treatment (35). Campylobacter continues to be one of the most common bacterial causes of diarrheal illness worldwide (36); many species primarily colonize the human oral cavity, and some strains can be translocated to the intestinal tract (37). Studies on human-hosted Campylobacter species strongly suggest that Campylobacter concisus plays a role in the development of inflammatory bowel disease (37), and 50.9% of Campylobacter ASVs were reliably identified as *C. concisus* in this study. Campylobacter gracilis was the second most common species, making up to 39.4%, and is a newly recognized species that is commonly found in the oral microbiota and has been associated with periodontal diseases and pleuropulmonary infections. These results indicated that the same set of microbial taxa were associated with the development and progression of NSCLC, and the biomarkers for DM might be acquired early.

Together, these results indicated that the sputum microbiota contributed more than the gut microbiota in patient stratification. In most cases, the sputum microbiota alone was sufficient for decent model performance.

### Altered pathways in sputum and gut microbial communities.

To evaluate functional alterations in the gut and sputum microbiota in NSCLC, we predicted the functional profiles for all samples based on their microbial compositions using PICRUSt2 (38).

We next identified significantly altered MetaCyc pathways (referred to as “marker pathways” below) between the Control and NSCLC groups for sputum and fecal samples separately. In total, we obtained 14 and 7 marker superpathways in sputum and the gut, respectively. Among the sputum marker superpathways, we found that 11 pathways increased and 3 decreased in the NSCLC group (Fig. S5A). The NSCLC group was enriched with pathways associated with menaquinol and demethylmenaquinol biosynthesis, purine and pyrimidine biosynthesis, and heme biosynthesis. In contrast, the pathways related to acidogenic fermentation and thiazole biosynthesis decreased in the NSCLC group. Among the 7 marker pathways in the gut, we found that 6 pathways increased and 1 decreased in the NSCLC group (Fig. S5B). The NSCLC group was enriched with pathways associated with fatty acid elongation and purine and pyrimidine biosynthesis. In contrast, the pathways related to peptidoglycan degradation decreased in the NSCLC group. Although the functional capacity of microbiota was inferred from PICRUSt2, these results might indicate the potential metabolic reprogramming in the sputum and gut microbiotas during the development of lung cancer, and metagenomic sequencing is needed to provide more evidence in the future.

### Pseudomonas aeruginosa, a species implicated in infections, was enriched in patients with brain metastasis.

**FIG 5:**
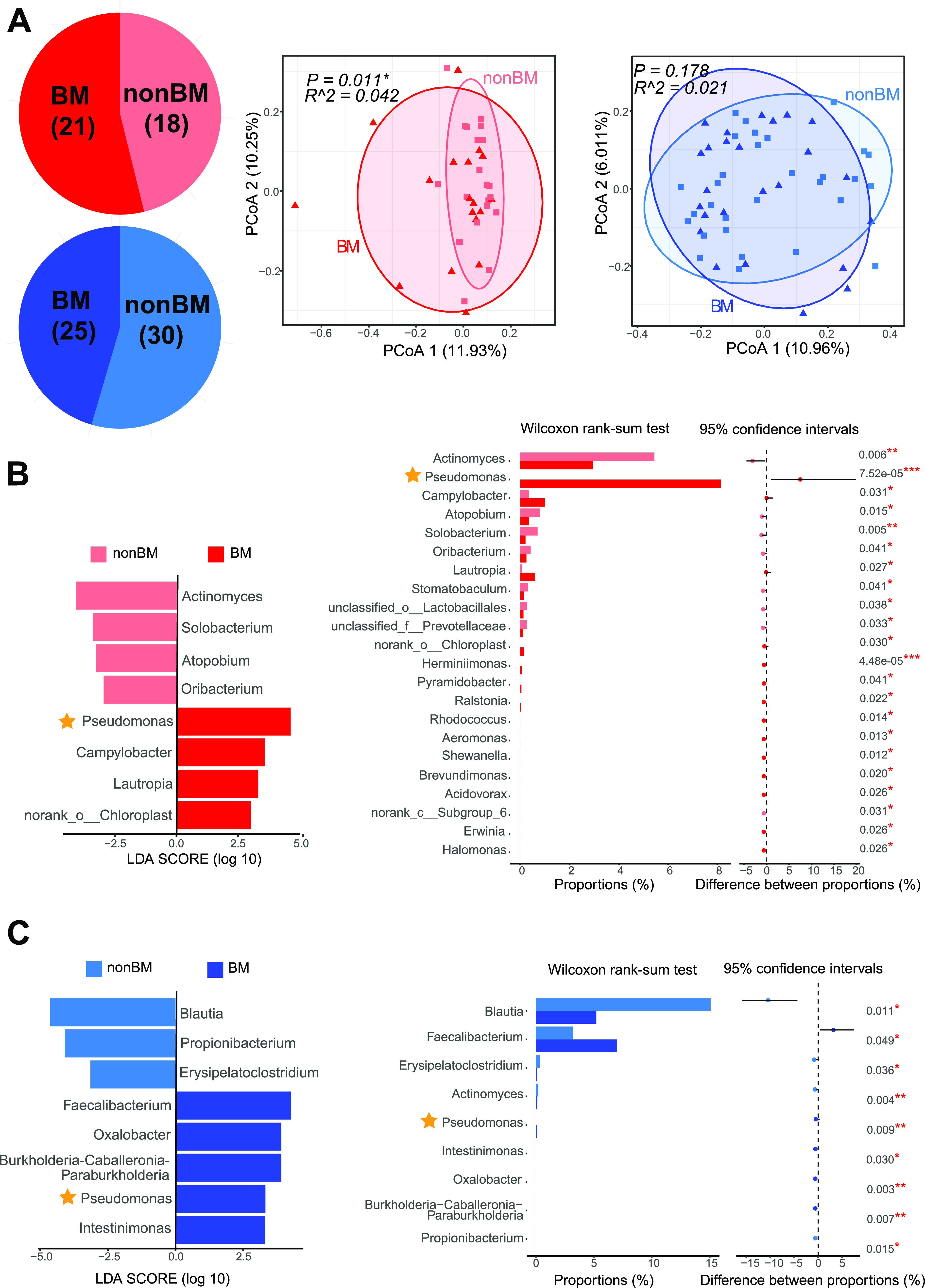
Patients with brain metastasis differed significantly from other distant metastasis in microbial profiles of the sputum and feces. (A) Numbers of sputum (red) and gut (blue) brain metastasis samples (left); BM, NSCLC patients in stage IV with brain metastasis; non-BM, stage IV NSCLC patients without brain metastasis. A principal-coordinate analysis showed differences in beta-diversity between BM and non-BM in sputum (middle) but not in the gut (right). LEfSe (left) analysis and Wilcoxon rank sum test (right) of differentially abundant microbial biomarkers between BM and non-BM in sputum (B) and the gut (C). Level of significance: ***, *P < *0.001; **, *P < *0.01; *, *P < *0.05; NS, *P ≥ *0.05. The star indicates that the genus Pseudomonas was significantly different in abundance.

**FIG 6:**
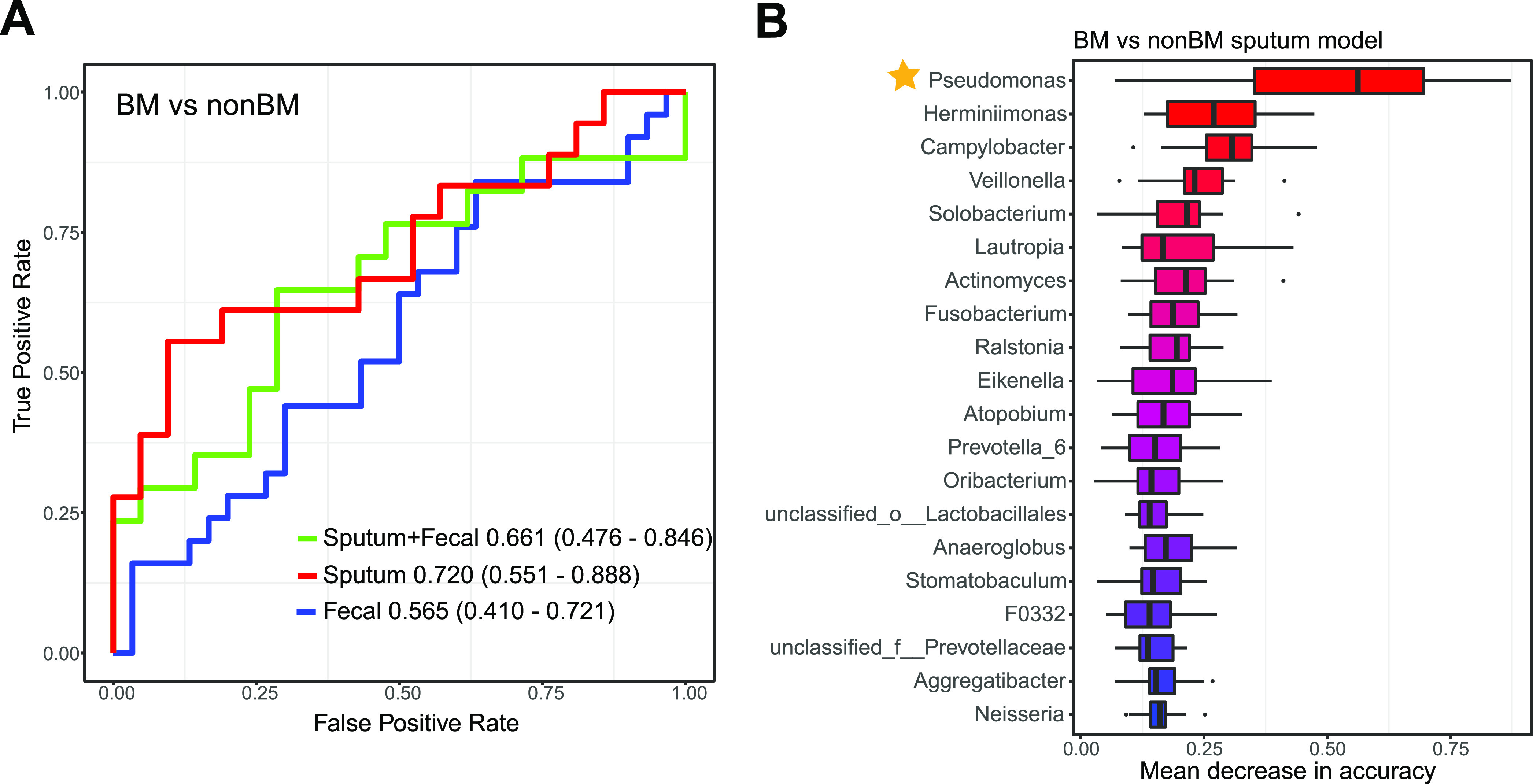
Brain metastasis classification based on taxonomic profiles of the sputum, gut, and both. (A) The classification performance using the relative abundance of genera as the area under the ROC curve between BM and non-BM. (B) The top 20 genera important to the sputum model ranked by the median values of 1,000 repeats. Box plots were used here to demonstrate the medians and distributions of these values. The star indicates that the genus Pseudomonas was significantly different in abundance using LEfSe and Wilcoxon analysis.

Brain metastasis (BM) represents the deadliest form of distant metastasis of NSCLC. To identify putative microbial biomarkers that are capable of distinguishing BM from other types of distant metastasis, we divided stage IV patients into two groups: the BM group (18 sputum samples and 25 fecal samples) and the non-BM group (21 sputum samples and 30 fecal samples) (Fig. 5A, left). As shown in Fig. 5A, in the sputum microbiota, we found significantly different beta-diversities (*P = *0.011; middle) between the two groups, while there was no significant difference in fecal microbiota (*P = *0.178; right). Thus, the dysbiosis of sputum microbiota was in stronger association with brain metastasis of NSCLC than the gut microbiota. We next performed LEfSe analysis and a Wilcoxon rank sum test to identify potential microbial biomarkers between BM and non-BM groups (Fig. 5B and C). Several differentially abundant genera were identified, including Pseudomonas and *Actinomyces* in sputum and *Blautia* and Pseudomonas in feces. Pseudomonas was highly abundant in the sputum of the BM group (∼8.14%) but was not detectable in the non-BM group, whose relative abundance was close to zero (Fig. 6B, right). Pseudomonas was also not detectable in any other disease stages or healthy controls. Pseudomonas was also significantly enriched in fecal samples of the BM group (with a relative abundance of ∼0.47%) and was not detectable in other fecal samples.

We then generated the distinguishing BM and non-BM models using the sputum microbiota, gut microbiota, and mixed microbiota separately. As shown in Fig. 6A, we found that the sputum microbiota performed best in the BM and non-BM group comparison. We also examined the top-ranking taxa in sputum, fecal, and mixed models. As shown in Fig. S6, there were more sputum-derived genera than gut-derived genera. Only three gut-derived genera were among the top 20 in the BM versus non-BM mixed model. Again, we found that Pseudomonas was the most important genus to sputum and mixed models between BM and non-BM (Fig. 6B; Fig. S6). Thus, Pseudomonas is significantly associated with brain metastasis in sputum. Pseudomonas consists of a group of aerobic, Gram-negative rod-shaped bacteria (1) that are associated with many human diseases but are relatively rare in the healthy gut (see https://gmrepo.humangut.info/species/286 for an overview of their prevalence and abundances in gut microbiota associated with human health and diseases [39]). According to the SPINGO tool, which assigns 16S rRNA gene sequencing reads to distinct taxa with confidence scores, most of the Pseudomonas ASVs (88.9%) could be reliably identified as Pseudomonas aeruginosa (see Materials and Methods). P. aeruginosa is one of the major causes of nosocomial infections worldwide (3) and is often associated with long-term wounds, pneumonia (4), chronic obstructive lung diseases (40), cystic fibrosis explanted lung (5), bronchiectasis (41), and chronic destroyed lung disease due to tuberculosis (40). Its roles in brain metastasis need to be further explored.

## DISCUSSION

We believe that the present study is the first to investigate alterations of both sputum (as a proxy for lung) and gut microbiotas on the development and metastasis of NSCLC. The results of our study suggest that lung microbiota may play major roles in the development of NSCLC, the dysbiosis of which could accurately stratify patients from healthy controls, while distant metastasis (DM) was associated with both sputum and gut microbiota dysbiosis. We further identified a prominent microbial biomarker for brain metastasis (BM).

In recent years, growing evidence has linked alterations in the lung or gut microbiota to LC or NSCLC. However, the relative importance of the lung and gut microbiotas to the development of NSCLC are still unclear. In addition, their alterations along with DM of NSCLC have not been characterized. Therefore, in this study, we assembled a cohort of patients diagnosed with NSCLC, including those suffering from DM (stage IV), and collected both sputum and fecal samples. We delineated the microbial community structure by 16S rRNA gene sequencing. The sputum and gut microbiota differed significantly in terms of alpha-diversity and beta-diversity, regardless of health status and disease stages. Surprisingly, sputum microbiota had significantly higher richness (taxon count) and evenness than gut microbiota, indicating unappreciated microbial complexity in the respiratory systems and critical roles in related diseases.

We built machine learning models to evaluate the relative importance of sputum and gut microbiota in patient stratification. We found that both sputum and gut microbiota dysbiosis contributed significantly to discriminating metastatic from nonmetastatic patients, while the sputum microbiota performed the best in discriminating stage I to III patients from DM patients. In most cases, the sputum microbiota alone or even a small number of important microbes was sufficient for decent model performance. These results highlighted the potential of using both sputum and gut microbiota in disease diagnosis.

By comparing healthy controls of matching demographic and clinical characteristics, we identified microbial biomarkers that showed significant abundance differences between subject groups. Not surprisingly, many of the identified biomarkers were either previously associated with other diseases (42, 43) or known to induce inflammation and/or interact with host immunity (26, 27, 44, 45). For example, the genera *Atopobium*, *Eggerthella*, and *Olsenella* (Fig. 3C), belonging to the family *Coriobacteriaceae*, have been implicated in the development of various clinical pathologies, including abscesses (27), periodontitis (28), intestinal diseases, and tumors (29, 30). *Atopobium* was the third most important genus to the I_III versus IV mixed model (Fig. 4F). Similarly, a genus *Filifactor*, which was the most important genus in the mixed model, was significantly enriched in healthy patients; it is known that some species of *Filifactor* are members of the human lung microbiome (46).

We identified that Pseudomonas aeruginosa was associated with brain metastasis (BM); P. aeruginosa was highly abundant in BM patients compared with other NSCLC patients as well as other distant metastatic patients and was exclusively found in sputum. P. aeruginosa is found in many diseases and is often associated with long-term wounds; its role in BM should be further experimentally determined.

Despite the strengths of our study, there were four limitations. First, we did not perform sequencing on negative controls. However, we took necessary procedures to avoid contaminations (for example, the use of sterilized materials and instruments throughout), and we would expect minimal contaminations. Even if there were any, we would expect similar contaminations in all samples because both sputum and fecal samples were taken from the same environment (i.e., the hospital). Thus, contaminations would only have very limited effects on our results and conclusions. Second, only a limited number of subjects was enrolled, which could limit the predictive performance of our patient stratification models; better machine learning models would have been possible with more subjects and deeper coverage of metagenomics sequencing data. Third, although easier to obtain and to monitor regularly, the sputum microbiota could be slightly different from that of the lung and could be contaminated by the oral microbiota. However, contaminations could be easily minimized by mouthwash before sputum collection and controlled for by sequencing the saliva samples from the same patients. Fourth, the exact roles of the gut and lung microbiotas in NSCLC and metastasis need to be further illustrated. Further experiments are needed to investigate their relative contributions by removing one at a time, and metagenomic sequencing is needed to provide more evidence on the functional capacity of the microbiota.

## MATERIALS AND METHODS

### Study design and sample collection.

We recruited in total 121 participants, including 87 newly diagnosed treatment-naive NSCLC patients of various stages and 34 healthy volunteers. Ninety-six sputum (66 NSCLC and 30 healthy) and 114 stool (85 NSCLC and 29 healthy) samples were collected, among which both sputum and stool samples were collected from 91 participants (65 NSCLC and 26 healthy; see Table S1 in the supplemental material). NSCLC patients were recruited in the Cancer Center, Union Hospital, Tongji Medical College, Huazhong University of Science and Technology, China. Healthy relatives of these patients were recruited as healthy controls. The criteria for selecting controls were as follows: good physical status and no significant respiratory or alimentary conditions. Patients who recently took drugs that were known to affect the gut microbiota, such as antibiotics (47), metformin (48), statin (49), and proton pump inhibitors (50), were excluded. NSCLC diagnosis was established according to histological criteria. The clinical stage of NSCLC was determined following the 8th American Joint Committee on Cancer guidelines; patients were classified into four distinct disease stages (i.e., from I to IV), in which stage IV referred to distant metastasis. No distant metastasis to any regions of the intestines was collected in this study.

The main exclusion criteria were as follows: less than 18 years of age, any antibiotic therapy within the previous 1 month, known chronic obstructive pulmonary disease, pneumoconiosis, silicosis or any other diseases of the respiratory system, known diabetes mellitus, hypertension and other basic diseases, and inability to give written informed consent. This study was approved by the Ethical Committees of the Cancer Center, Union Hospital, Tongji Medical College, Huazhong University of Science and Technology (2018-S271) and was registered with ClinicalTrials.gov (identifier NCT03454685). All participants provided written informed consent before sample donation.

All fecal and spontaneous sputum samples were obtained after NSCLC diagnosis and before the patients received treatment. The sputum samples were collected from participants in the morning in sterilized containers. To avoid possible contamination from the oral microbiota, participants were required to rinse their mouths with sterilized saline buffer three times (51, 52). Healthy controls who might not have sputum for the first time were revisited until a sputum sample could be taken. These samples were immediately stored at −80°C. Demographic and clinical data, including smoking status, gender, age, body mass index (BMI), disease stage, and lung cancer pathology, were obtained from each participant.

### DNA extraction.

Bacterial DNA was extracted from the fecal and sputum samples using the OMEGA-soil DNA kit (Omega Bio-Tek, USA) according to the manufacturer’s instructions. The quality of DNA was measured using a NanoDrop 2000 spectrophotometer (Thermo Scientific, USA) as well as 1% agarose gel electrophoresis. Bacterial DNA was immediately stored at −80°C until further analysis.

### 16S rRNA amplification and sequencing.

Bacterial DNA was isolated from fecal and sputum samples as previous described. DNA libraries covering the V3-V4 hypervariable regions of the bacterial 16S rRNA gene were constructed using the FastPfu polymerase (TransGen, China) according to the manufacturer’s instructions. We used the primer set composed of 338F (5′-ACTCCTACGGGAGGCAGCAG-3′) and 806R (5′-GGACTACHVGGGTWTCTAAT-3′), which was designed to amplify the V3-V4 hypervariable region. All PCR products were purified with an AxyPrep DNA gel extraction kit (Axygen Biosciences, USA) and quantified using a QuantiFluor-ST (Promega, USA) according to the manufacturer’s instructions. Paired-end sequencing with a read length of 300 bp (PE300) of the PCR amplification products was performed on an Illumina MiSeq platform (Illumina, USA) by Majorbio Bio-Pharm Technology Co., Ltd. (Shanghai, China). Sequence data have been deposited to the NCBI SRA database under the NCBI BioProject ID PRJNA576323.

### Sequencing data analysis and taxonomic assignment.

Overall read quality was checked for each sample using FastQC (version 0.11.5). After Trimmomatic (version 0.35) (53), reads with quality less than 30 or length less than 100 bp were removed from subsequent analysis. The DADA2 (version 1.18.0) (54) pipeline was used to filter the sequencing reads and construct ASV tables without any sequence clustering. The taxonomy classification database used was GreenGenes version 13.8. Taxa with relative abundances below 0.0001 (0.01%) were excluded from further analysis. All analyses were performed at the genus level because there was no significant difference found in diversity analysis at the ASV level. The taxonomy classification at the species level was identified using SPINGO (55), which is a rapid species classifier for microbial amplicon sequences.

### Statistics analysis.

Patient characteristics were expressed as mean ± standard deviation (SD) and compared using χ^2^ tests or independent samples *t* tests as appropriate. Statistical analyses were performed using SPSS V.19.0 for Windows (Statistical Product and Service Solutions, Chicago, IL, USA).

Diversity analyses were performed using the R package “Vegan.” In alpha-diversity analysis, Wilcoxon rank sum tests were used to compare two groups, while an analysis of variance (ANOVA) with *post hoc* Tukey honestly significant difference (HSD) test was used to compare multiple groups. A principal-coordinate analysis and an Adonis test were performed based on Bray-Curtis distance, and pairwise differences between groups were assessed using the pairwise.adonis function adjusted for FDR correction.

LEfSe analysis (56) and Wilcoxon rank sum tests (57) were used to identify differentially abundant genera between subject groups. The LEfSe analysis was used to generate the relative abundance table using R package “microbiomeMarker” downloaded from GitHub (https://github.com/yiluheihei/microbiomeMarker) (options: transform = “identity”; norm = “CPM”; kw_cutoff = 0.05; lda_cutoff = 2; bootstrap_n = 30; wilcoxon_cutoff = 0.05).

For distinguishing the NSCLC patients and controls based on the taxonomic composition of their sputum and gut microbes, the random forest binary model was built. All data were loaded into R version 4.0 and analyzed. To get a robust classifier and avoid the bias of one-time splits, the createMultiFolds algorithm from the “caret” package was used to split the data set 10 times and generated the 10-time 10-fold cross-validation random forest model and feature importance using the SIAMCAT package (34). The data set was randomly divided into two parts: the training set and the test set, whereby the training set accounted for 90% of the samples. The recursive feature elimination (RFE) function of the “caret” package was used to do the feature selection applying random forest modeling and 10-fold cross validation with 10 times repeat, corresponding to the number of features that should be retained in the updated model in the range of 2 to 50. The features with low overall abundance (0.001) were filtered, and then the reserved features were normalized by the “log.std” method. In the DAM model, the check.assocation function was used to calculate the significance of enrichment and metrics of association. To plot the results of the evaluation, the function model.evaluation.plot was used to show the receiver operating characteristic (ROC) curves for the different resample runs as well as the mean ROC curve. From the 10-time repeated 10-fold cross-validation, the feature weights were summarized as contributions to assess the importance of features to the model. For external validation, the random forest model was applied to the testing data set, and the prediction results were summarized to the ROC value.

The functional profiles for all samples were predicted using PICRUSt2 (38).

### Data availability.

Sequencing data are available and have been deposited to the NCBI SRA project under the NCBI BioProject ID PRJNA576323. All scripts and data have been uploaded to GitHub (https://github.com/GaoData/NSCLC). Methods, including statements of data availability and additional references, are available at the publisher’s website.
