# Supplementary material for: Alterations of the Human Lung and Gut Microbiomes in Non-Small Cell Lung Carcinomas and Distant Metastasis
Source: Microbiol Spectr. 2021 Nov 17;9(3):e00802-21. doi: 10.1128/Spectrum.00802-21 (PMC8597645; doi:10.1128/Spectrum.00802-21)
Supplement: SUPPLEMENTAL FILE 1 — Supplemental material. Download Spectrum.00802-21-s0001.pdf, PDF file, 5.1 MB [file spectrum.00802-21-s0001.pdf]

Supplementary Figure 1. Taxonomic distribution of sputum and gut microbiota at the phylum and genus level. The heatmap plot of relative abundance at the phylum level in healthy controls and distinct disease stage groups of sputum (A) and the gut (B). CON: healthy controls; NSCLC: patients; I_III: patients with stages of I to III; DM: patients with distant metastasis (also referred to as stage IV). Disease stages were assigned according to the 8th American Joint Committee on Cancer guidelines. (C) Comparisons of phyla between sputum with the gut. There were 10 genera shared by the gut and sputum. (D) The top ten genera with high relative abundance in healthy controls and distinct disease stage groups of the sputum (upper panel) and gut (lower panel).

Supplementary figure 2. Sputum and gut microbiota differed significantly in terms of alpha diversities in healthy controls (A) and NSCLC (B). Shannon diversity index (middle panel) and Simpson index were significantly lower in feces compared to sputum. Wilcoxon rank-sum test was used to compare between groups. Level of significance: *** $P<0.001$; ** $P<0.01$; * $P<0.05$; NS. $P \geqslant 0.05$.

Supplementary figure 3. The alpha- and beta- diversities of sputum and gut microbiota. (**A**) Alpha diversity of sputum dysbiosis in pairwise comparisons, no significance was found in Evenness index. ANOVA with post-hoc Tukey HSD test was used to compare between groups. Level of significance: *** $P<0.001$; ** $P<0.01$; * $P<0.05$; NS. $P\geq 0.05$. (**B**) Significant differences were found in beta-diversity between controls and NSCLC in sputum, indicating that dysbiosis of sputum microbiota was associated with lung cancer development. Conversely, applying similar analyses to

fecal samples, neither alpha-diversities (**C-F**) nor beta-diversity (**G**) was significantly different.

Supplementary figure 4. The top-ranked genera of the mixed RFE models for each disease stage. The genera were ranked by the robustness of 1000 repeats; therefore, boxplots were used here to demonstrate the means and distributions of these values. **(A)** Control vs NSCLC; **(B)** Control vs I_III; (**C**) CON vs DM and (**D**) I_III vs DM. Red boxes: sputum-derived genera; blue boxes: gut-derived genera. Please consult Table 2 for details on the model performance.

Supplementary Figure 5. Pathway analysis of sputum (A) and fecal (B). The significant MetaCyc pathways between controls vs NSCLC groups.

Supplementary figure 6. The top twenty genera ranked according to their importance to the mixed models in BM vs nonBM. Red boxes: sputum-derived genera; blue boxes: gut-derived genera. Star demonstrated the genus *Pseudomonas* was significantly different in abundance.

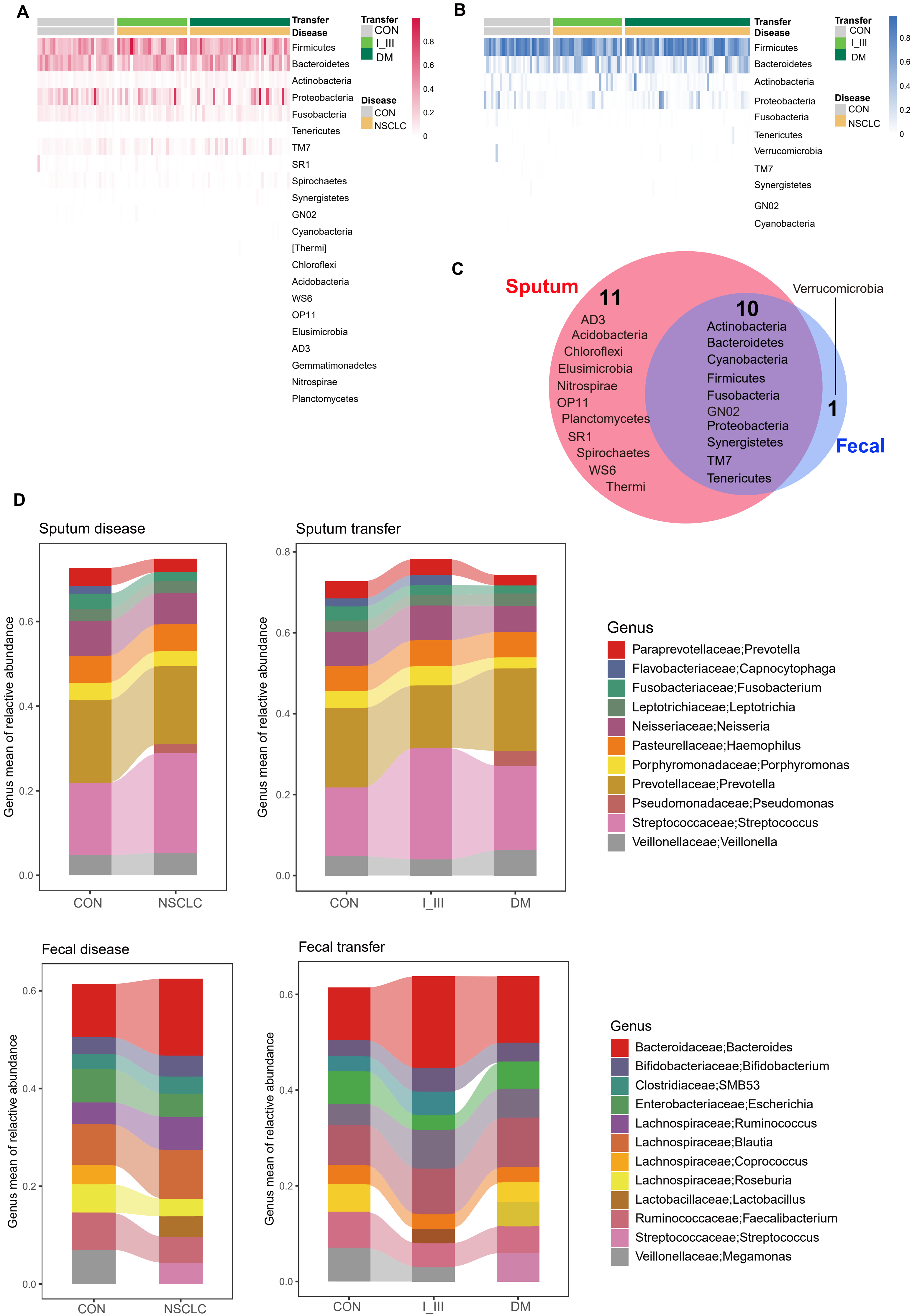

A
Transfer
Disease
Firmicutes
Bacteroidetes
Actinobacteria
Proteobacteria
Fusobacteria
Tenericutes
TM7
SR1
Spirochaetes
Synergistetes
GN02
Cyanobacteria
[Thermi]
Chloroflexi
Acidobacteria
WS6
OP11
Elusimicrobia
AD3
Gemmatimonadetes
Nitrospirae
Planctomycetes
Transfer
CON
I_III
DM
Disease
CON
NSCLC
0.8
0.6
0.4
0.2
0
B
Transfer
Disease
Firmicutes
Bacteroidetes
Actinobacteria
Proteobacteria
Fusobacteria
Tenericutes
Verrucomicrobia
TM7
Synergistetes
GN02
Cyanobacteria
Transfer
CON
I_III
DM
Disease
CON
NSCLC
0.8
0.6
0.4
0.2
0
C
Sputum
11
AD3
Acidobacteria
Chloroflexi
Elusimicrobia
Nitrospirae
OP11
Planctomycetes
SR1
Spirochaetes
WS6
Thermi
10
Actinobacteria
Bacteroidetes
Cyanobacteria
Firmicutes
Fusobacteria
GN02
Proteobacteria
Synergistetes
TM7
Tenericutes
Verrucomicrobia
1
Fecal
D
Sputum disease
Genus mean of relactive abundance
0.6
0.4
0.2
0.0
CON
NSCLC
Sputum transfer
Genus mean of relactive abundance
0.8
0.6
0.4
0.2
0.0
CON
I_III
DM
Genus
Paraprevotellaceae;Prevotella
Flavobacteriaceae;Capnocytophaga
Fusobacteriaceae;Fusobacterium
Leptotrichiaceae;Leptotrichia
Neisseriaceae;Neisseria
Pasteurellaceae;Haemophilus
Porphyromonadaceae;Porphyromonas
Prevotellaceae;Prevotella
Pseudomonadaceae;Pseudomonas
Streptococcaceae;Streptococcus
Veillonellaceae;Veillonella
Fecal disease
Genus mean of relactive abundance
0.6
0.4
0.2
0.0
CON
NSCLC
Fecal transfer
Genus mean of relactive abundance
0.6
0.4
0.2
0.0
CON
I_III
DM
Genus
Bacteroidaceae;Bacteroides
Bifidobacteriaceae;Bifidobacterium
Clostridiaceae;SMB53
Enterobacteriaceae;Escherichia
Lachnospiraceae;Ruminococcus
Lachnospiraceae;Blautia
Lachnospiraceae;Coprococcus
Lachnospiraceae;Roseburia
Lactobacillaceae;Lactobacillus
Ruminococcaceae;Faecalibacterium
Streptococcaceae;Streptococcus
Veillonellaceae;Megamonas

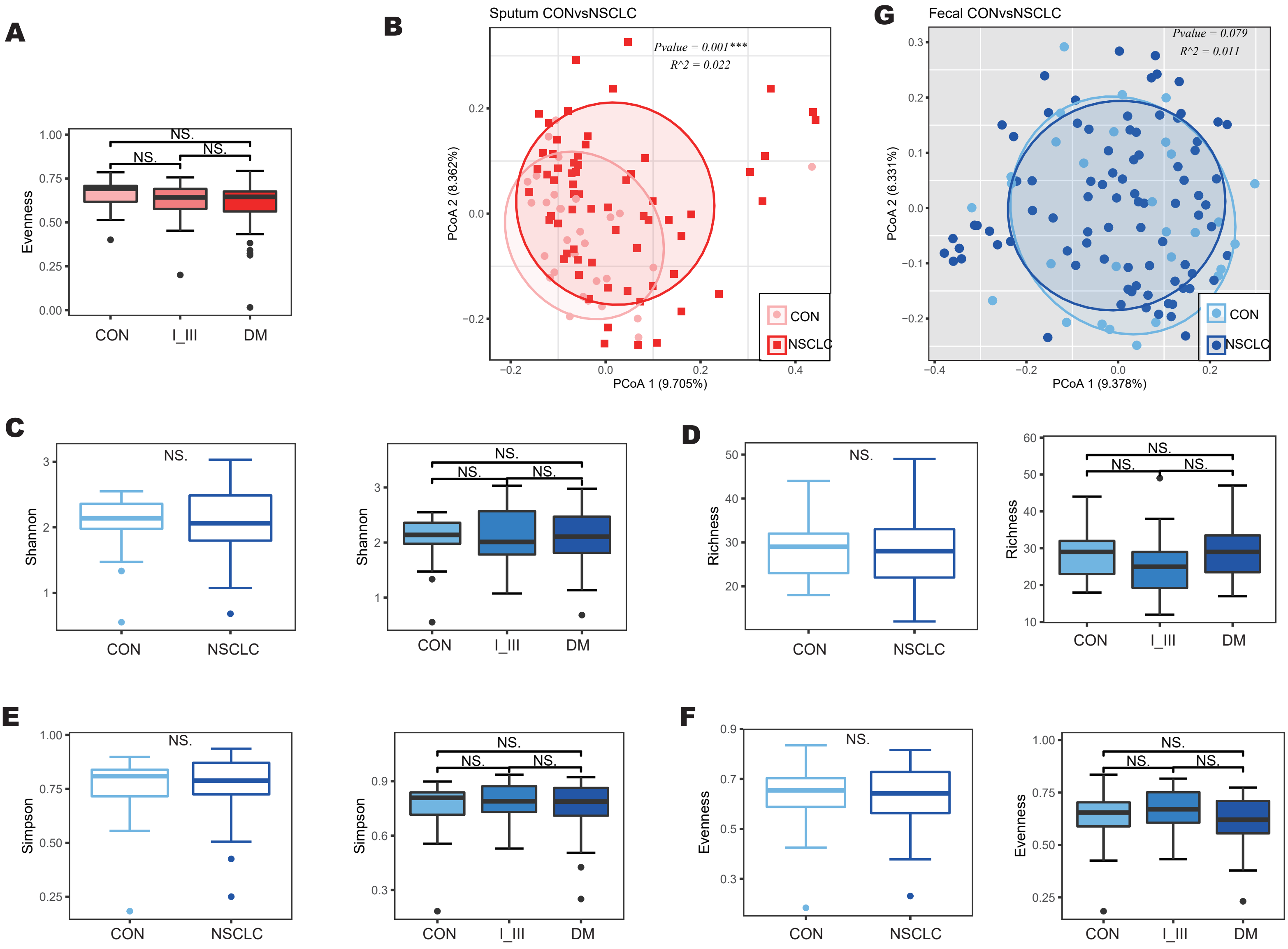
A
Evenness
NS.
NS.
NS.
1.00
0.75
0.50
0.25
0.00
CON
I_III
DM
B
Sputum CONvsNSCLC
Pvalue = 0.001***
R^2 = 0.022
PCoA 2 (8.362%)
PCoA 1 (9.705%)
0.2
0.0
−0.2
0.4
CON
NSCLC
G
Fecal CONvsNSCLC
Pvalue = 0.079
R^2 = 0.011
PCoA 2 (6.331%)
PCoA 1 (9.378%)
0.3
0.2
0.1
0.0
−0.1
−0.2
−0.4
CON
NSCLC
C
Shannon
NS.
CON
NSCLC
NS.
NS.
NS.
CON
I_III
DM
D
Richness
NS.
CON
NSCLC
NS.
NS.
NS.
CON
I_III
DM
E
Simpson
NS.
1.00
0.75
0.50
0.25
CON
NSCLC
NS.
NS.
NS.
0.9
0.6
0.3
CON
I_III
DM
F
Evenness
NS.
0.9
0.7
0.5
0.3
CON
NSCLC
NS.
NS.
NS.
1.00
0.75
0.50
0.25
CON
I_III
DM

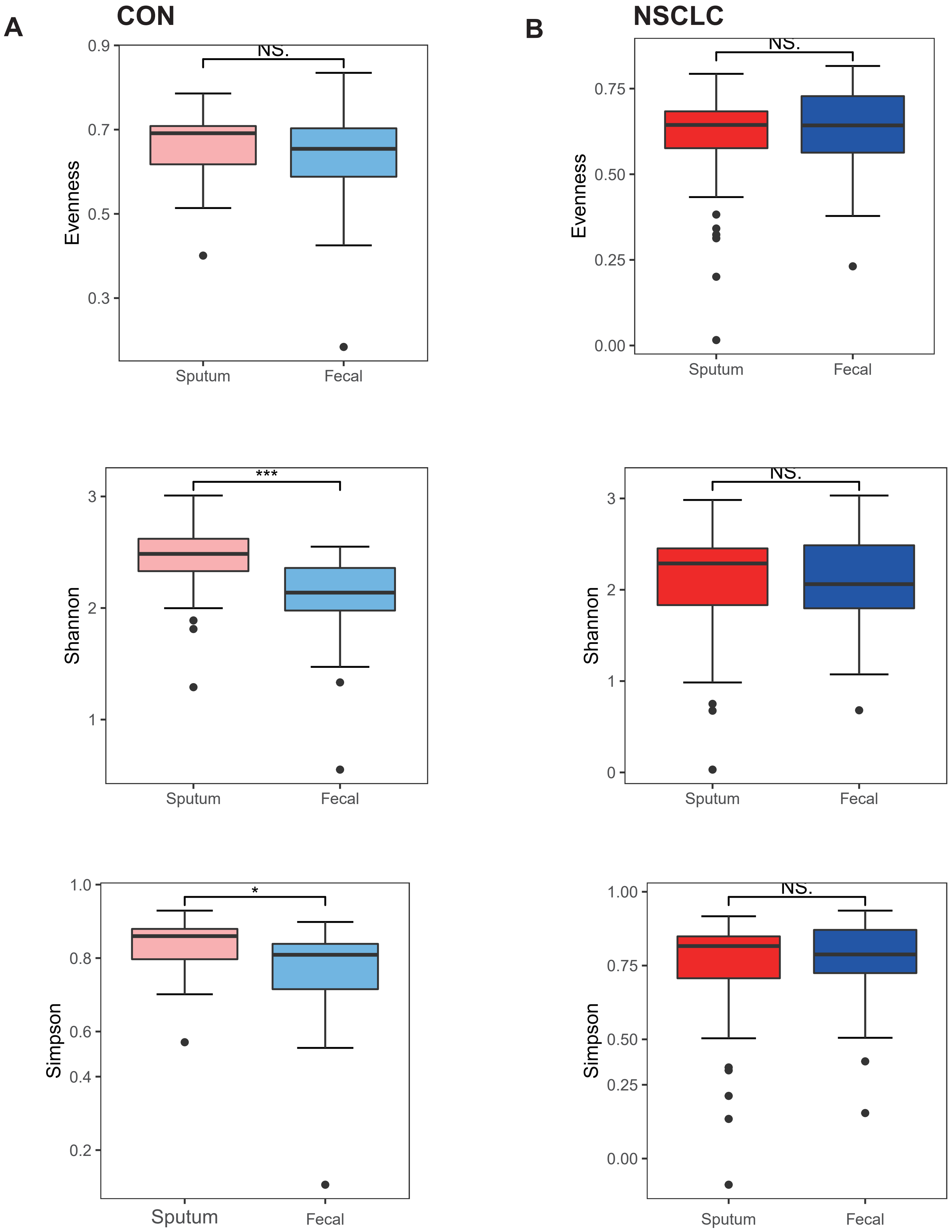

A
CON
B
NSCLC
NS.
***
*
Evenness
Shannon
Simpson
Sputum
Fecal
0.9
0.7
0.5
0.3
0.75
0.50
0.25
0.00
3
2
1
0
1.0
0.8
0.6
0.4
0.2
1.00

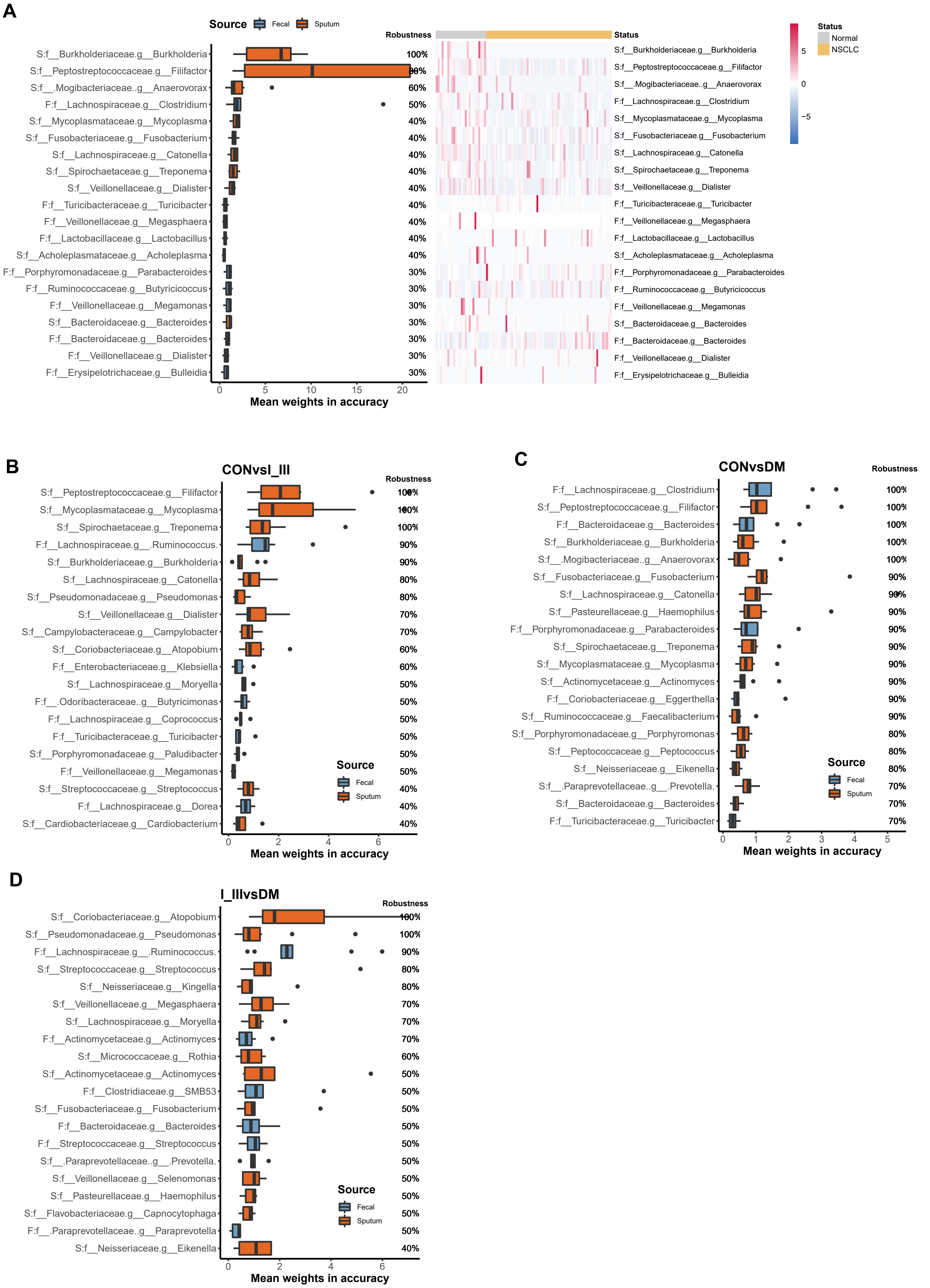

A
Source
Fecal
Sputum
Robustness
Status
Normal
NSCLC
5
0
−5
S:f__Burkholderiaceae.g__Burkholderia 100%
S:f__Peptostreptococcaceae.g__Filifactor 90%
S:f__.Mogibacteriaceae..g__Anaerovorax 60%
F:f__Lachnospiraceae.g__Clostridium 50%
S:f__Mycoplasmataceae.g__Mycoplasma 40%
S:f__Fusobacteriaceae.g__Fusobacterium 40%
S:f__Lachnospiraceae.g__Catonella 40%
S:f__Spirochaetaceae.g__Treponema 40%
S:f__Veillonellaceae.g__Dialister 40%
F:f__Turicibacteraceae.g__Turicibacter 40%
F:f__Veillonellaceae.g__Megasphaera 40%
F:f__Lactobacillaceae.g__Lactobacillus 40%
S:f__Acholeplasmataceae.g__Acholeplasma 40%
F:f__Porphyromonadaceae.g__Parabacteroides 30%
F:f__Ruminococcaceae.g__Butyricicoccus 30%
F:f__Veillonellaceae.g__Megamonas 30%
S:f__Bacteroidaceae.g__Bacteroides 30%
F:f__Bacteroidaceae.g__Bacteroides 30%
F:f__Veillonellaceae.g__Dialister 30%
F:f__Erysipelotrichaceae.g__Bulleidia 30%
0 5 10 15 20
Mean weights in accuracy
B
CONvsI_III
Robustness
S:f__Peptostreptococcaceae.g__Filifactor 100%
S:f__Mycoplasmataceae.g__Mycoplasma 100%
S:f__Spirochaetaceae.g__Treponema 100%
F:f__Lachnospiraceae.g__.Ruminococcus. 90%
S:f__Burkholderiaceae.g__Burkholderia 90%
S:f__Lachnospiraceae.g__Catonella 80%
S:f__Pseudomonadaceae.g__Pseudomonas 80%
S:f__Veillonellaceae.g__Dialister 70%
S:f__Campylobacteraceae.g__Campylobacter 70%
S:f__Coriobacteriaceae.g__Atopobium 60%
F:f__Enterobacteriaceae.g__Klebsiella 60%
S:f__Lachnospiraceae.g__Moryella 50%
F:f__.Odoribacteraceae..g__Butyricimonas 50%
F:f__Lachnospiraceae.g__Coprococcus 50%
F:f__Turicibacteraceae.g__Turicibacter 50%
S:f__Porphyromonadaceae.g__Paludibacter 50%
F:f__Veillonellaceae.g__Megamonas 50%
S:f__Streptococcaceae.g__Streptococcus 40%
F:f__Lachnospiraceae.g__Dorea 40%
S:f__Cardiobacteriaceae.g__Cardiobacterium 40%
Source
Fecal
Sputum
0 2 4 6
Mean weights in accuracy
C
CONvsDM
Robustness
F:f__Lachnospiraceae.g__Clostridium 100%
S:f__Peptostreptococcaceae.g__Filifactor 100%
F:f__Bacteroidaceae.g__Bacteroides 100%
S:f__Burkholderiaceae.g__Burkholderia 100%
S:f__.Mogibacteriaceae..g__Anaerovorax 100%
S:f__Fusobacteriaceae.g__Fusobacterium 90%
S:f__Lachnospiraceae.g__Catonella 90%
S:f__Pasteurellaceae.g__Haemophilus 90%
F:f__Porphyromonadaceae.g__Parabacteroides 90%
S:f__Spirochaetaceae.g__Treponema 90%
S:f__Mycoplasmataceae.g__Mycoplasma 90%
S:f__Actinomycetaceae.g__Actinomyces 90%
F:f__Coriobacteriaceae.g__Eggerthella 90%
S:f__Ruminococcaceae.g__Faecalibacterium 90%
S:f__Porphyromonadaceae.g__Porphyromonas 80%
S:f__Peptococcaceae.g__Peptococcus 80%
S:f__Neisseriaceae.g__Eikenella 80%
S:f__.Paraprevotellaceae..g__.Prevotella. 70%
S:f__Bacteroidaceae.g__Bacteroides 70%
F:f__Turicibacteraceae.g__Turicibacter 70%
Source
Fecal
Sputum
0 1 2 3 4 5
Mean weights in accuracy
D
I_IIIvsDM
Robustness
S:f__Coriobacteriaceae.g__Atopobium 100%
S:f__Pseudomonadaceae.g__Pseudomonas 100%
F:f__Lachnospiraceae.g__.Ruminococcus. 90%
S:f__Streptococcaceae.g__Streptococcus 80%
S:f__Neisseriaceae.g__Kingella 80%
S:f__Veillonellaceae.g__Megasphaera 70%
S:f__Lachnospiraceae.g__Moryella 70%
F:f__Actinomycetaceae.g__Actinomyces 70%
S:f__Micrococcaceae.g__Rothia 60%
S:f__Actinomycetaceae.g__Actinomyces 50%
F:f__Clostridiaceae.g__SMB53 50%
S:f__Fusobacteriaceae.g__Fusobacterium 50%
F:f__Bacteroidaceae.g__Bacteroides 50%
F:f__Streptococcaceae.g__Streptococcus 50%
S:f__.Paraprevotellaceae..g__.Prevotella. 50%
S:f__Veillonellaceae.g__Selenomonas 50%
S:f__Pasteurellaceae.g__Haemophilus 50%
S:f__Flavobacteriaceae.g__Capnocytophaga 50%
F:f__.Paraprevotellaceae..g__Paraprevotella 50%
S:f__Neisseriaceae.g__Eikenella 40%
Source
Fecal
Sputum
0 2 4 6
Mean weights in accuracy

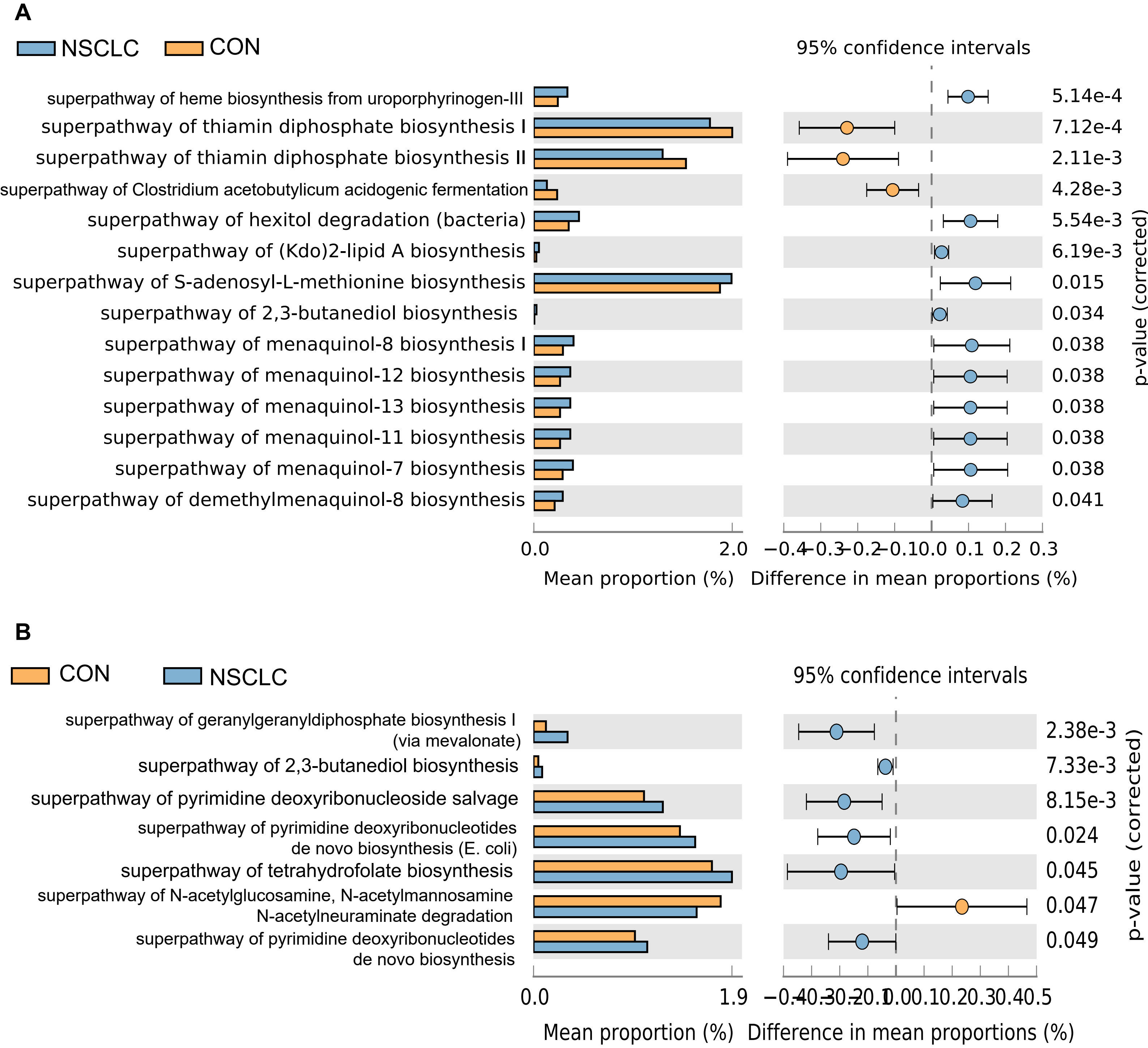

A
NSCLC
CON
95% confidence intervals
superpathway of heme biosynthesis from uroporphyrinogen-III
superpathway of thiamin diphosphate biosynthesis I
superpathway of thiamin diphosphate biosynthesis II
superpathway of Clostridium acetobutylicum acidogenic fermentation
superpathway of hexitol degradation (bacteria)
superpathway of (Kdo)2-lipid A biosynthesis
superpathway of S-adenosyl-L-methionine biosynthesis
superpathway of 2,3-butanediol biosynthesis
superpathway of menaquinol-8 biosynthesis I
superpathway of menaquinol-12 biosynthesis
superpathway of menaquinol-13 biosynthesis
superpathway of menaquinol-11 biosynthesis
superpathway of menaquinol-7 biosynthesis
superpathway of demethylmenaquinol-8 biosynthesis
5.14e-4
7.12e-4
2.11e-3
4.28e-3
5.54e-3
6.19e-3
0.015
0.034
0.038
0.038
0.038
0.038
0.038
0.041
p-value (corrected)
0.0
2.0
−0.4 −0.3 −0.2 −0.1 0.0 0.1 0.2 0.3
Mean proportion (%)
Difference in mean proportions (%)
B
CON
NSCLC
95% confidence intervals
superpathway of geranylgeranyldiphosphate biosynthesis I (via mevalonate)
superpathway of 2,3-butanediol biosynthesis
superpathway of pyrimidine deoxyribonucleoside salvage
superpathway of pyrimidine deoxyribonucleotides de novo biosynthesis (E. coli)
superpathway of tetrahydrofolate biosynthesis
superpathway of N-acetylglucosamine, N-acetylmannosamine N-acetylneuraminate degradation
superpathway of pyrimidine deoxyribonucleotides de novo biosynthesis
2.38e-3
7.33e-3
8.15e-3
0.024
0.045
0.047
0.049
p-value (corrected)
0.0
1.9
−0.4 −0.3 −0.2 −0.1 0.0 0.1 0.2 0.3 0.4 0.5
Mean proportion (%)
Difference in mean proportions (%)

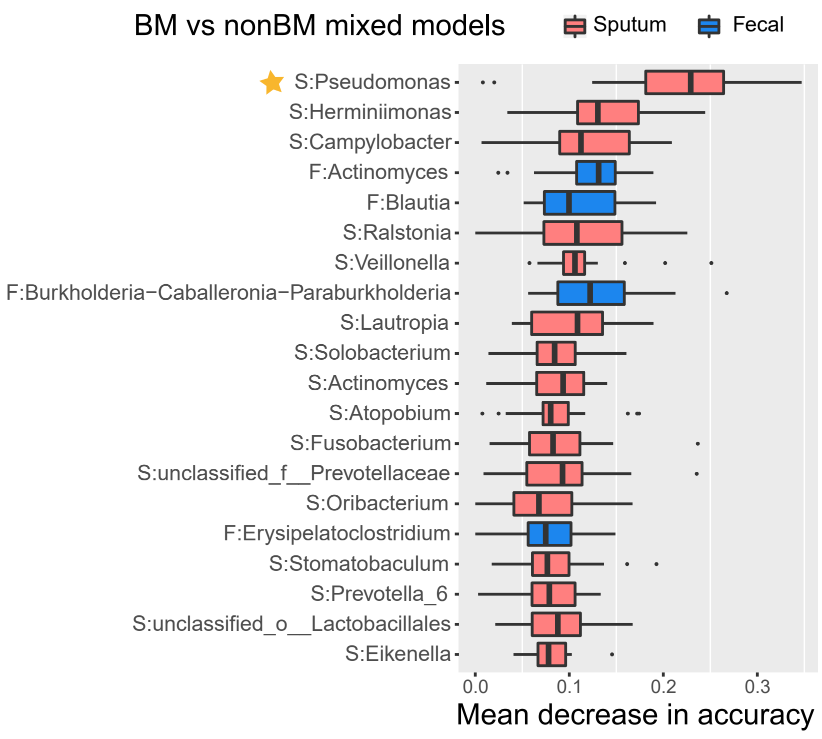

BM vs nonBM mixed models
Sputum
Fecal
S:Pseudomonas
S:Herminiimonas
S:Campylobacter
F:Actinomyces
F:Blautia
S:Ralstonia
S:Veillonella
F:Burkholderia−Caballeronia−Paraburkholderia
S:Lautropia
S:Solobacterium
S:Actinomyces
S:Atopobium
S:Fusobacterium
S:unclassified_f__Prevotellaceae
S:Oribacterium
F:Erysipelatoclostridium
S:Stomatobaculum
S:Prevotella_6
S:unclassified_o__Lactobacillales
S:Eikenella
0.0
0.1
0.2
0.3
Mean decrease in accuracy
